# Aroma Volatile Compounds from Two Fresh Pineapple Varieties in China

**DOI:** 10.3390/ijms13067383

**Published:** 2012-06-14

**Authors:** Liang-Yong Zheng, Guang-Ming Sun, Yu-Ge Liu, Ling-Ling Lv, Wen-Xiu Yang, Wei-Feng Zhao, Chang-Bin Wei

**Affiliations:** 1 South Subtropical Crop Research Institute, Chinese Academy of Tropical Agricultural Science, Zhanjiang 524091, Guangdong, China; E-Mails: zhengliangyong46@163.com (L.-Y.Z.); gm-sun@163.com (G.-M.S.); liuyugehb@126.com (Y.-G.L.); lulingling1234@21cn.com (L.-L.L.); 2 Yunnan Vocational College of Tropical Crops, Pu’er 665000, Yunnan, China; E-Mails: xiaoyangyang111@126.com (W.-X.Y.); zwf9721zwf9721@163.com (W.-F.Z.)

**Keywords:** pineapple, aroma volatile compounds, odor active values (OAVs), GC/MS

## Abstract

Volatile compounds from two pineapples varieties (Tainong No.4 and No.6) were isolated by headspace solid phase microextraction (HS-SPME) and identified and quantified by gas chromatography-mass spectrometry (GC/MS). In the Tainong No. 4 and No. 6 pineapples, a total of 11 and 28 volatile compounds were identified according to their retention time on capillary columns and their mass spectra, and quantified with total concentrations of 1080.44 μg·kg^−1^ and 380.66 μg·kg^−1^ in the Tainong No.4 and No. 6 pineapples, respectively. The odor active values (OAVs) of volatile compounds from pineapples were also calculated. According to the OAVs, four compounds were defined as the characteristic aroma compounds for the Tainong No. 4 pineapple, including furaneol, 3-(methylthio)propanoic acid methyl ester, 3-(methylthio)propanoic acid ethyl ester and δ-octalactone. The OAVs of five compounds including ethyl-2-methylbutyrate, methyl-2-methylbutyrate, 3-(methylthio)propanoic acid ethyl ester, ethyl hexanoate and decanal were considered to be the characteristic aroma compounds for the Tainong No. 6 pineapple.

## 1. Introduction

Native to Central and South America, pineapple (*Ananas comosus* L. Merr.) is widely distributed in tropical regions such as Hawaii, India, Malaysia, Philippines, Thailand and several provinces of China. In recent years, the pineapple has become one of the most popular exotic fruits in demand [1]. Pineapple varieties are plentiful, but only a few of the leading types are sold commercially. Due to its attractive sweet flavor, pineapple is widely consumed as both fresh and canned fruits, as well as in processed juices and as an ingredient in exotic foods.

The volatile components of pineapples have been studied extensively. More than 280 compounds are known to be involved in generating the characteristic pineapple flavor [2–6].

The aroma volatile compounds that produce the characteristic aroma of pineapple depend on the pineapple varieties [7,8], different areas where the pineapple crop is grown [6], different seasons [9], different stages of ripening [10,11], development of the fruit [10,12], storage conditions [12], postharvest storage [13] and flesh position [14,15]. These parameters have been the subject of extensive studies showing that the volatile compounds found in pineapple include a variety of esters, lactones, acids, hydrocarbons, sulfur-containing compounds and carbonyl compounds.

Although much work has been done, discussion of the aroma quality and the quantity of each constituent and their contributions of each of these constituents to the fresh, sweet aroma of pineapple grown in China is still scarce. Using odor values calculated from odor threshold and concentration, In 1989, Takeoka *et al*. [3] reported that the important contributors to the fresh pineapple aroma are 4-hydroxy-2,5-dimethyl-3[2H]-furanone (HDF), methyl-2-methylbutanoate, ethyl-2-methylbutanoate, ethyl acetate, ethyl hexanoate, ethyl butanoate, ethyl-2-methylpropanoate, methyl hexanoate, and methyl butanoate.

Pineapple is the third most important tropical fruit in mainland China. The two varieties were released in Chinese Taipei in the 1980s, and introduced to mainland China, in areas such as Fujian province and Hainan province. Pineapples of the Tainong varieties are consumed fresh. The aromas of Tainong No.4 and Tainong No. 6 pineapple are different, although pineapple varieties were all selected from hybrid progeny of “smooth cayenne♀” × “Shen wan♂”. The aim of the present work was to identify and determine the compounds responsible for the characteristic aromas of the two pineapple varieties cultivated in China and to illustrate differences in aroma compounds between the two varieties.

## 2. Results and Discussion

### 2.1. The Volatile Aroma Compounds of Two Pineapple Varieties

#### 2.1.1. The Volatiles of Two Varieties

The aroma volatile compounds have been extensively studied for 60 years, and many researchers have focused on these compounds [16–18]. To date, more than 280 volatile compounds have been identified among the aroma volatiles of pineapple, but few volatile compounds that had been identified contributed to the pineapple aroma [19].

The volatile compounds observed in pineapples are shown in Table 1. The analysis of the SPME extract of pineapple indicated the presence of 33 volatile compounds. Nineteen of these compounds were esters, five were terpenens, three lactones, one ketone, one alcohol, one aldehyde, and two acids in the two pineapple varieties. From the yield of aroma volatiles, esters represented the main group of aroma volatile compounds in the pineapple, followed by lactones and terpenes. Esters were found to be the most abundant pineapple volatiles. Takeoka *et al*. reported that ethyl hexanoate and methyl hexanoate had the highest concentrations and contributed to the pineapple aroma [4]. Recently, Wei *et al*. and Akioka *et al*. also reported that esters were the major volatile compounds in the pineapple [15,20].

#### 2.1.2. Differences in Esters in the Two Varieties of Pineapple

The total concentrations of esters were 326.26 μg·kg^−1^ and 914.05 μg·kg^−1^ for the Tainong No. 6 and No. 4 pineapples, respectively. Five volatile compounds were simultaneously present in the two pineapple varieties. These compounds included methyl hexanoate, 3-(methylthio)propanoic acid methyl ester, 3-(methylthio)propanoic acid ethyl ester, methyl octanoate and methyl decanoate. Table 1 showed the experimental determination of the volatile compounds, and the compound with the highest concentration was 3-(methylthio)propanoic acid ethyl ester at 78.06 μg·kg^−1^, followed by ethyl octanoate and 3-(methylthio)propanoic acid methyl ester in the Tainong No. 6 pineapple variety. In the Tainong No. 4 pineapple, 3-(methylthio)propanoic acid methyl ester had the highest concentration at 622.49 μg·kg^−1^, followed by methyl octanoate (142.25 μg·kg^−1^) and methyl hexanoate (67.75 μg·kg^−1^). In the pineapple pulp of French Polynesia, 3-(methylthio)propanoic acid methyl ester and 3-(methylthio)propanoic acid ethyl ester were identified with respective concentrations of 1140 μg·kg^−1^ and 150 μg·kg^−1^ [6]. The pineapple of French Polynesia showed higher concentrations of these compounds than the Tainong No. 6 and No. 4 pineapples. Methyl octanoate had the highest amount (1496 μg·kg^−1^) [6], higher than the value for this compound found in Tainong No. 6 and No. 4 pineapples. A concentration of methyl octanoate of 197 μg·kg^−1^ was found in the pineapples of French Polynesia [6]. This value was much higher than the concentrations of this aroma compound found in the present work for any of the Tainong No. 4 and No.6 varieties of pineapple.

#### 2.1.3. Differences in Lactones in the Two Varieties

The family of lactone compounds represented 5.89% and 5.87% of the total aroma volatiles in the Tainong No. 6 and No. 4 pineapples, respectively. These concentrations were lower than relative proportion of 11.5% found in French Polynesian pineapples [6], but the Tainong pineapples showed higher values for the lactones than the values found by Umano *et al*. (4.6% for green fruit and 2.8% for ripe fruit) [5]. The relative concentration of γ-octalactone and δ-octalactone were 140 μg·kg^−1^ and 48 μg·kg^−1^ in French Polynesian pineapples [6]. In our study, γ-octalactone was detected in the Tainong No. 6 pineapple at a concentration of 12.49 μg·kg^−1^, and δ-octalactone was found in the Tainong No. 4 pineapple at a concentration of 63.40 μg·kg^−1^.

#### 2.1.4. Differences in Ketones and Terpenes in the Two Varieties

For the ketone family of compounds, 2,5-dimethyl-4-hydroxy-3(2*H*)-furanone (furaneol) was found at a relatively higher concentration of 76.47 μg·kg^−1^ in Tainong No.4, but was not found in the Tainong No.6 pineapple.

The terpene compounds were found to be present in Tainong No. 6 and Tainong No. 4 at lower concentrations. Five terpenoid compounds were found in Tainong No. 6, but only one terpenoid compounds (α-muurolene) was found in the Tainong No. 4 pineapple. α-Muurolene was found in essential oils from mango flowers, but at a lower concentration [21].

### 2.2. The Characteristic Aroma Compounds of Pineapple

#### 2.2.1. The Characteristic Aroma Compounds in the Two Varieties of Pineapple

The characteristic aroma compounds were determined by odor activity values (OAVs), the ratio of the concentration of the compound to the odor threshold [17]. When the OAVs is >1 for a compound, that compound would play a critical role in fruit flavor and is considered to be the characteristic aroma compounds for the fruits. Orthonasal odor threshold in water and odor activity value for several volatile compounds found in different pineapple varieties are shown in Table 2.

Five compounds were defined as the characteristic aroma compounds based on the OAV of compounds found in the Tainong No. 6 pineapple, where ethyl-2-methylbutyrate showed the highest OAV of 3706.67, followed by methyl-2-methylbutyrate with an OAV of 77.92. The compound 3-(methylthio)propanoic acid ethyl ester also contributed to the aroma of pineapple as one of the thioester compounds because its OAVs was higher than 1.

According to their OAVs, four compounds were defined as the characteristic aroma compounds of the Tainong No. 4 pineapple. 2,5-Dimethyl-4-hydroxy-3(2*H*)-furanone (furaneol) had the highest OAV of 2549, and 3-(methylthio)propanoic acid methyl ester, 3-(methylthio)propanoic acid ethyl ester and δ-octalactone also had the OAVs higher than 1, so these values also made us consider that the listed compounds contributed to the characteristic aroma of the Tainong No. 4 pineapple.

Furaneol was considered one of the most important characteristic aroma compounds in pineapples, based on the higher OAVs. Furaneol was one of the most important flavor compounds in strawberries, due to the low odor threshold [23,24], and was identified for the first time as a natural aroma component in pineapples [25].

#### 2.2.2. Difference of Characteristic Aroma Compounds in Two Varieties

Based on the OAVs of the aroma compounds listed, the difference between characteristic aroma compounds was illustrated for the two varieties by means of OAVs. The main aroma family of Tainong No.6 was the same as the main aroma family of Tainong No. 4, namely, esters and ketones. Although 3-(methylthio)propanoic acid ethyl ester was present in the two varieties, the other characteristic aroma compounds of the two varieties were different.

Takeoka *et al*. (1989; 1991) [3,4] had determined the odor threshold of 3-(methylthio)propanoic acid methyl ester and 3-(methylthio)propanoic acid ethyl ester to be 180 μg·kg^−1^ and 7 μg·kg^−1^. Their respective concentrations indicated their great odor contribution to Tainong No. 4 and No. 6 pineapples according to their respective OAVs. Two thioesters, including 3-(methylthio)propanoic acid methyl ester and 3-(methylthio)propanoic acid ethyl ester, were the characteristic aroma compounds in Tainong No. 4, whereas only one thioester was founded in the Tainong No.6 pineapple, namely, 3-(methylthio)propanoic acid ethyl ester.

Methyl-2-methylbutyrate and ethyl-2-methylbutyrate were detected in the Tainong No. 6 pineapple. Their odor thresholds were 0.25 μg·kg^−1^ and 0.006 μg·kg^−1^, respectively [3,4]. Their OAVs were calculated and allowed us to consider those two compounds as the major characteristic aromas of the Tainong No. 6 pineapple. Ethyl-2-methylbutyrate is the main aroma in apples [26]. The smell of a typical apple-like aroma [27,28] could be why Tainong No. 6 is called ‘apple’ pineapple.

Decanal and ethyl butanoate are often the most important contributors to the flavor of orange juice [18]. Decanal has a OAV of 16.1, and is considered to be one of the key aroma volatile compounds contributing to the characteristic aroma of the Tainong No. 6 pineapple, as it is one of its aldehyde compounds.

Characteristic aroma compounds of the two varieties were different from those of the cayenne pineapple. In the cayenne pineapple, the characteristic aroma compounds were ethyl 2-methylbutanoate, ethyl hexanoate, 2,5-dimethyl-4-hydroxy-3(2*H*)-furanone (DMHF), decanal, ethyl 3-(methylthio)propionate, ethyl butanoate, and ethyl (E)-3-hexenoate in the pulp. In the pineapple core, the main compounds were found to be ethyl 2-methylbutanoate, ethyl hexanoate and DMHF [15].

## 3. Experimental Section

### 3.1. Fruit Samples

The pineapple varieties that were tested are Tainong No. 4 and No. 6. Three fruits of each variety were used for analysis, obtained from the Southern Subtropical Crops Research Institute of the Chinese Academy of Tropical Agricultural Science in Zhanjiang of Guangdong province.

### 3.2. Initial Quality Characteristics

Initial quality characteristics were assessed by total soluble solids and color (Table 3). Total Soluble Solids (TSS) were measured with an Atago Hand Refractometer (ATAGO, master-m, Japan) on opposite sides of each fruit. The color of skin and pulp, expressed as Hunter L, a, and b values, was measured on the most and least colored parts of three fruits from each variety using a colorimeter (Tintometer, Lovibond RT100, UK).

### 3.3. Isolation of Volatile Compounds

After the removal of the skin, the pulp was stirred and mixed by a food processor. The volatile compounds of the pulp were extracted by solid-phase microextraction: 10 g of homogenated samples was introduced into a glass vial with a magnetic stirring bar (8 × 25 mm), and the vials were well capped with a 20 mm diameter aluminum seal and a Teflon septum and kept under constant vigorous stirring. The SPME syringe was then manually inserted into the headspace of the vial with a fiber coating of 65 μm polydimethylsiloxane/divinylbenzene (PDMS/DVB) purchased from Supelco. The aroma compounds were extracted at 25 °C for 40 min. After extraction, the SPME fiber was placed into the injector of the GC/MS instrument.

### 3.4. Identification of the Volatile Compounds

An Agilent 6890 gas chromatograph coupled to an Agilent 5975 N mass spectrometer (Agilent, Santa Clara, CA, USA) was utilized for this analysis. When extraction was completed, the SPME fiber coating was immediately introduced into the GC injection port at 250 °C and maintained for 3 min. Separation of analytes was achieved with a HP-5MS capillary column (30 m × 0.25 mm × 0.25 μm film thickness) (HP, Palo Alto, CA, USA) under an oven temperature program as follows: 40 °C initially, then increased to 120 °C at a rate of 3 °C/min, then increased to 200 °C at a rate of 5 °C/min and held for 10 min. Purified helium (purity 99.999%) was used as the carrier gas at 1.0 mL/min constant flow rate. The mass spectrometer was operated in scan mode from *m/z* 35 to 335, with 70 eV electron ionization at 230 °C, quadrupole at 150 °C.

Compounds were identified by matching their mass spectra and the retention time with standards and the NIST2005 database library. The concentration represented by each major volatile peak was determined by using octadecane as internal standard. The characteristic aroma compounds were defined by the odor activity value (OAV) in aroma volatiles of pineapple [15]. The OAV was calculated using the ratio of the concentration of each component to the odor threshold in water [22,30].

## 4. Conclusions

Although the two varieties of pineapple were selected from the same hybrid match, the aroma profiles of the two varieties were different. These results indicated that the aromatic character of pineapple was a result of the interaction of esters, sulfur compounds, terpenes and other compounds. A total of 11 and 28 volatile compounds were identified and quantified in the Tainong No. 4 and No. 6 pineapples, respectively. According to the OAVs, four compounds were found to be the characteristic aroma compounds for the Tainong No. 4 pineapple, including furaneol, 3-(methylthio)propanoic acid methyl ester, 3-(methylthio)propanoic acid ethyl ester and δ-octalactone. Five compounds with OAVs above 1 in the Tainong No. 6 pineapple were considered to be the characteristic aroma compounds: ethyl-2-methylbutyrate, methyl-2-methylbutyrate, 3-(methylthio)propanoic acid ethyl ester, ethyl hexanoate and decanal.

## Figures and Tables

**Table 1:** The volatile aroma compounds of the two pineapple varieties.

Classification	Compounds Name	Content/(μg·kg^−1^)	
		Tainong No.6	Tainong No. 4
Esters	Butanoic acid, 2-methyl-, methyl ester	19.48	-
	Butanoic acid, 2-hydroxy-2-methyl-, methyl ester	-	32.27
	Butanoic acid, 2-methyl-, ethyl ester	22.24	-
	Hexanoic acid, methyl ester	4.71	67.75
	Hexanoic acid, ethyl ester	8.35	-
	2,4-Hexadienoic acid, methyl ester	1.37	-
	3-(Methylthio)propanoic acid methyl ester	32.94	622.49
	Butane-2,3-diyl diacetate	12.23	-
	3-(Methylthio)propanoic acid ethyl ester	78.06	32.96
	Octanoic acid, methyl ester	20.52	142.25
	Ethyl 3-hydroxyhexanoate	8.61	-
	Benzoic acid, ethyl ester	9.68	-
	Octanoic acid, ethyl ester	46.21	-
	Benzeneacetic acid, ethyl ester	0.73	-
	Dodecanoic acid, methyl ester	18.28	16.33
	Methyl cinnamate	6.54	-
	Decanoic acid, ethyl ester	19.96	-
	Ethyl cinnamate	15.38	-
	Dodecanoic acid, ethyl ester	0.97	-
Total		326.26	914.05

Terpenes	(±)-Dictyopterene A	15.61	-
	4,9-Muuroladiene	3.64	-
	α-Muurolene	3.60	12.03
	(−)-Alloaromadendrene	1.26	-
	1,6-Cyclodecadiene	0.52	-
Total		24.63	12.03

Lactones	γ-Octalactone	12.49	-
	5-Hydroxyoctanoic acid lactone	9.93	-
	δ-Octalactone	-	63.40
Total		22.42	63.4

Ketones	2,5-Dimethyl-4-hydroxy-3(2*H*)-furanone	-	76.47
Total		-	76.47

Alcohol	2,3-Dimethyl-undec-1-en-3-ol	-	6.25
Total		-	6.25

Aldehydes	Decanal	1.61	-
Total		1.61	-

Acids	Octanoic Acid	-	8.24
	Ethyl (±)-3-acetoxybutyrate	3.90	-
Total		3.90	8.24

Alkenes	3,4-Dimethoxystyrene	1.84	-
Total		1.84	-

Grand total		**380.66**	**1080.44**

-: Not detected.

**Table 2:** The characteristic aroma compounds and odor activity values of pineapples.

Compounds	Odor Threshold/(μg·kg^−1^) a	Odor Activity Value		Odor Quality

		Tainong No.6	Tainong No.4	
3-(Methylthio)propanoic acid methyl ester	180 [6]	0.18	3.46	meaty, oniony, fruity at low level
3-(Methylthio)propanoic acid ethyl ester	7 [6]	11.15	4.71	Meaty, oniony, pineapple-like at low level
Butanoic acid, 2-methyl-, methyl ester	0.25 [3,4,22]	77.92	-	Fruity, apple-like
Butanoic acid, 2-methyl-, ethyl ester	0.006 [3,4,22]	3706.67	-	Fruity
Hexanoic acid, methyl ester	70 [22]	0.067	0.97	Fruity, ester-like
Hexanoic acid, ethyl ester	0.76 [22]	10.99	-	Apple peel-like, fruity
2,5-Dimethyl-4-hydroxy-3(2*H*)-furanone	0.03 [22]	-	2549	Sweet, pineapple and caramel-like
Caryophyllene	64 [22]	-	-	Bitter
Octanoic acid, methyl ester	200 [22]	0.10	0.71	Fruity, citrus-like
Decanal	0.1 [22]	16.1	-	
Octanal	8 [17]	-	-	Citrus, fatty
δ-Octalactone	0.400 [17]	-	158.5	Coconut-like
Decanoic acid, ethyl ester	6300 [22]	0.003	-	

a odor thresholds in water.

**Table 3:** Initial quality characteristics of two varieties.

Category		Varieties	
	
		Tainong No.6	Tainong No.4
TSS (%)		13.25 ± 0.27	16.80 ± 1.10
Hunter color			
Skin	L *	27.66 ± 2.44	28.77 ± 1.05
	a *	0.41 ± 0.62	1.68 ± 0.56
	b *	−9.02 ± 0.91	−8.18 ± 0.60
Pulp	L *	28.26 ± 1.98	37.65 ± 3.74
	a *	–0.24 ± 0.62	1.40 ± 0.58
	b *	−7.04 ± 1.00	−3.07 ± 1.56

The L* data represent the whiteness of the color with 100 representing white and 0 representing black. The a* data represent red and green: a positive value indicates a red color with +60 being the maximum, whereas a negative value indicates a green color with −60 being the maximum. Similarly, b* represents yellow and blue, (+) being yellow and (−) indicating blue [29]. The skin and pulp were both measured.
